# Carcinogenicity prediction via multi-task learning of cross-organ representations with attention mechanisms

**DOI:** 10.1093/bib/bbag296

**Published:** 2026-06-04

**Authors:** Yunju Song, Hwan Choi, Sunyong Yoo

**Affiliations:** Department of Intelligent Electronics and Computer Engineering, Chonnam National University, 77, Yongbong-ro, Buk-gu, Gwangju, 61186, Republic of Korea; Department of Mechanical and Aerospace Engineering, University of Central Florida, 12760 Pegasus Dr, Orlando, FL 32816, United States; Department of Intelligent Electronics and Computer Engineering, Chonnam National University, 77, Yongbong-ro, Buk-gu, Gwangju, 61186, Republic of Korea; R&D Center, MATILO AI Inc., 77, Yongbong-ro, Buk-gu, Gwangju, 61186, Republic of Korea

**Keywords:** cancer, carcinogenicity prediction, multi-task learning, graph attention network, attention mechanism, organ specificity

## Abstract

Cancer is caused by the uncontrolled growth and division of abnormal cells. In industrialized societies, chemical exposure is a leading cause of cancer. Since certain compounds induce cancer by damaging genes or affecting cellular metabolism, studying carcinogens is essential. However, previous studies used separate models for each organ and failed to capture carcinogenic features shared across organs, limiting generalization. Thus, this study developed a multi-task learning framework to predict organ-specific carcinogenicity in the liver, lung, stomach, and breast. This framework consisted of a shared layer and task-specific layers. The shared layer contains a graph attention network layer to make atom-level representations, along with parallel fully connected layers designed for each task combination. The resulting shared representations are passed to task-specific layers to predict organ-specific carcinogenicity. The training process followed stepwise learning, whereby the model was first trained using partially labeled data to capture cross-organ representations and determine initial weights. In the second step, fully labeled data for all organs were used for final training. The proposed multi-task model achieved superior performance in the liver, lung, and stomach tasks. Notably, it recorded the highest area under the receiver operating characteristic curve in the stomach task (0.7636), outperforming the single-task model (0.7055) and all comparative models (0.5527–0.7418). The highest area under the precision–recall curve was observed in the liver task (0.9646), surpassing the single-task model (0.9505) and all comparative models (0.9373–0.9621). We further analyzed molecules with high predicted carcinogenicity and identified critical substructures using an attention mechanism. This research can contribute to predicting organ-specific carcinogenicity of candidate chemicals in the early stages of drug development.

## Graphical Abstract

**Graphical Abstract:**
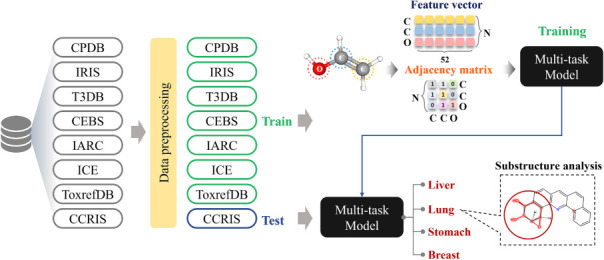

## Introduction

Cancer is a disease caused by the uncontrolled proliferation of cells and represents one of the leading causes of death worldwide, causing millions of deaths annually [1]. Cancer can be caused by environmental factors, with exposure to carcinogens being a major risk [2]. Humans in industrialized societies are continuously exposed to various chemicals; some are classified as carcinogens [3]. These carcinogenic chemicals have the potential to promote cancer through DNA damage or by disrupting cellular metabolic processes [4]. Consequently, the analysis of carcinogens is becoming increasingly important. Carcinogen evaluations have traditionally utilized *in vitro* and *in vivo* experimental assays [5–7]. *In vitro* assays have the advantages of not requiring animal experiments and reducing resources and time [8, 9]. However, these *in vitro* assays cannot account for the physiological effects of compounds on organs, as these assays are limited to isolated cellular environments [9]. In contrast, while *in vivo* assays can assess systemic effects, these are resource- and time-intensive. Moreover, the carcinogenicity of only a small proportion of chemical compounds has been tested using *in vitro* and *in vivo* methods [9, 10]. To address these limitations, numerous studies have sought to apply machine learning and deep learning approaches including *in silico* methods to solve a wide range of biological and medical problems [11–14]. These *in silico* methods represent an effective time and cost alternative to predict the carcinogenicity of many compounds [15].

Previous studies using *in silico* methods have utilized machine learning techniques such as support vector machines, random forest, and deep learning models [16–20]. Within these methods, it is important to select model structures that effectively capture the properties of compounds for accurate carcinogenicity prediction. The complex intramolecular connectivity information is a critical factor that determines the physicochemical properties, biological activity, toxicity, and pharmacological effects of compounds. Accordingly, various molecular representations have been explored. For example, sequence-based approaches such as Mol-BERT treat the Simplified Molecular Input Line Entry System (SMILES) as a chemical language and apply transformer-based pretraining to learn molecular representations from large-scale unlabeled compounds [21]. However, SMILES strings are linearized representations that may not fully preserve the topological connectivity among atoms [22]. In contrast, molecular graphs visually represent molecular structures and encode topological connectivity directly, without requiring complex transformations [23–25]. Thus, graph-based neural networks such as graph convolutional networks (GCNs) and graph attention networks (GATs) have recently been widely employed to predict molecular properties [26–29]. For instance, CarcGC, a GCN-based model, has been proposed for chemical carcinogenicity prediction [30]. However, GCNs aggregate information from neighboring nodes with the same weight to update the representation of each node, which may constrain the ability of this network to capture the critical atoms in molecular structures [31–33]. To overcome this limitation, a GAT assigns attention weights to relationships between nodes during information aggregation from neighbors, giving higher weights to important atoms [34, 35]. Accordingly, GAT-based methods have been actively applied to carcinogenicity prediction [36]. For example, DCAMCP employs GAT layers to process molecular graph features and combines them with molecular fingerprints through a capsule network to predict carcinogenicity [37]. However, most studies treat carcinogenicity as a single organ-agnostic outcome despite considerable variation in carcinogenic effects across organs. The carcinogenic effects of chemicals vary across organs and are closely related to organ-specific cancer mechanisms [38], as pharmacokinetics, metabolic activation, and detoxification capacity differ among organs, meaning certain compounds may induce cancer in some organs but not others [39, 40]. Consequently, models that do not account for organ specificity risk underestimating or overestimating the true carcinogenic risk of a given compound. Even in studies that attempted to incorporate organ specificity, some relied on statistical structural similarity [41]. In contrast, other studies analyzed associations among compounds, lesions, and organ-specific cancers using rule-based machine learning [42]. More importantly, these approaches used only organ-specific descriptors, limiting the ability to capture cross-organ representations of carcinogens and, consequently, restricting the generalizability of the models. To address these issues, multi-task learning is needed to determine the carcinogenicity of compounds in multiple organs simultaneously. This approach is effective because it has been shown to improve the generalization performance of models by learning various tasks simultaneously. Therefore, previous studies have applied multi-task learning to biological tasks such as predicting properties of compounds (e.g. side effects, toxicity, protein targets) and predicting aquatic toxicity or lethal doses in different species [43–46].

Based on previous biological approaches, we constructed a multi-task learning framework for carcinogenicity prediction that integrates both cross-organ representations and GAT-based modeling. This model uses three-task combination data in the first step and data from all tasks in the second. This approach enables the simultaneous extraction of generalized carcinogenicity patterns and organ-specific, thereby enhancing the efficiency of information utilization and improving predictive performance compared to building separate binary classification models [47, 48].

## Materials and methods

### Data collection

To construct the integrated database, we collected carcinogenicity evaluation data for various chemicals from eight sources, each providing comprehensive information on the relevant experiments.

The The Carcinogenic Potency Database (CPDB) was developed at the National Library of Medicine and the National Institutes of Health [49]. The CPDB contains the results of carcinogenicity bioassays for 1547 substances. This database provides information on the following variables, including species, sex, route of administration, target organ, and carcinogenicity status, and includes experimental results published in scientific papers and experimental results reported in technical reports by the National Cancer Institute (NCI)/National Toxicology Program (NTP).

The Chemical Carcinogenesis Research Information System (CCRIS) was developed at the NCI and comprises separate databases on carcinogenicity, mutagenicity, tumor promotion, and tumor suppression bioassay data for chemical substances [50]. This study uses the carcinogenicity bioassays database, which provides information on 1767 substances, including details on the species, sex, route of administration, tumor site, and carcinogenicity status.

The United States Environmental Protection Agency (EPA) Integrated Risk Information System (IRIS) includes human health risk assessment information for 738 chemicals commonly found in the environment [51]. The database provides detailed information on the route of administration, tumor site and type, toxicity values, and human carcinogenicity status of the chemical substances under investigation.

The Toxin and Toxin Target Database (T3DB) was developed by the Wishart Research Group [52]. The T3DB contains data on 3678 toxins and provides extensive information, including toxicity values, human carcinogenicity classification, and chemical characteristics of tissues and toxins. This study only used the all-toxin data from the T3DB.

The Chemical Effects in Biological Systems (CEBS), maintained by the National Institute of Environmental Health Sciences, is a public repository of toxicological study data and metadata generated by the NTP [53]. Organ-specific neoplasm occurrence data were obtained from the ‘Organ Sites with Neoplasia’ dataset, covering 228 compounds in this study.

The International Agency for Research on Cancer (IARC) database provides systematically curated records of tissue- and organ-specific tumor sites observed in both humans and experimental animals [54]. Tumor and tumor site information for Group 1 agents was utilized, with a focus on recognized carcinogens, contributing data for 30 compounds.

Integrated Chemical Environment (ICE) aggregates *in vivo* and *in vitro* cancer hazard classification data from multiple regulatory agencies, supplemented by detailed findings from NTP two-year cancer bioassays [55]. The cancer dataset was used as the primary source, covering 120 compounds.

The Toxicity Reference Database (ToxRefDB), a U.S. EPA-maintained resource of guideline-compliant *in vivo* study data, offers structured information on organ- and tissue-specific tumor incidence and dose–response relationships across a wide range of chemicals, covering 273 compounds in the present analysis [56].

### Data labeling

The CPDB contains information on SMILES data of compounds, target tissues, toxic dose 50% (TD50), and carcinogenicity experimental data. For the carcinogenicity experimental results, we removed data labeled ‘0’, which does not provide clear evidence of carcinogenicity, and data labeled ‘e’, which indicates uncertain evidence of carcinogenicity; data labeled ‘c’, ‘p’, ‘a’, and ‘+’ were classified as ‘carcinogenic positive’, while data labeled ‘-’ were classified as ‘carcinogenic negative’ (Table 1).

**Table 1:** Classification of carcinogenicity labels based on CPDB.

**CPDB data**	**CPDB results**	**Descriptions**	**Labels**
Literature-based data	+	This indicates that the author in the literature evaluated the tissue–tumor combination as being induced by the test agent.	Positive
	−	The author explicitly indicated that the test agent did not induce tumors at the specific site.	Negative
	0	The author provided no opinion or an ambiguous opinion on the carcinogenicity of the test agent.	Remove
NCI/NTP data	c	The test agent was evaluated as carcinogenic in the NCI/NTP Technical Report.	Positive
	p	There is some evidence of carcinogenicity.	Positive
	a	The tumors are associated with carcinogenicity, or the evidence is suggestive.	Positive
	e	There is equivocal evidence of carcinogenicity.	Remove
	0	NCI/NTP did not present an evaluation for this tissue–tumor combination, or the experiment was evaluated as inadequate.	Remove
	-	For NCI/NTP experiments without a ‘c’, ‘p’, ‘a’, or ‘e’ opinion, one site is denoted a ‘-’ opinion unless the experiment was inadequate.	Negative

The CCRIS contains information on SMILES of compounds, tumor site information, and carcinogenicity experimental results. The results of the carcinogenicity experiments are presented in a string format, such as ‘negative’, indicating no carcinogenicity in the test, ‘positive’ indicating carcinogenicity, and ‘equivocal’, indicating an ambiguous result. To ensure consistency within the dataset, we excluded data representing a certain percentage of tumors or unclear results. Only data classified as ‘positive’ or ‘negative’ were used. The ‘positive’ data were labeled as ‘carcinogenic positive’, and the ‘negative’ data as ‘carcinogenic negative’.

The IRIS database comprises numerous pieces of information, including the CAS number, assessment type (cancer/non-cancer), critical effect tumor type (toxicity type caused by compound exposure), and toxicity value. This study initially mapped the SMILES data provided by PubChem based on the CAS number. Meanwhile, based on assessment type data, compounds were labeled as ‘carcinogenic positive’ when classified as ‘cancer’ and ‘carcinogenic negative’ when classified as ‘non-cancer’. Furthermore, data were extracted on the tissues in which carcinogenicity was reported through the critical effect tumor type field.

The T3DB comprises CAS numbers, toxicity data, carcinogenicity data, and data regarding the affected tissue. As with the IRIS database, the SMILES data provided by PubChem were initially mapped based on the CAS number. The carcinogenicity evaluation was conducted using the carcinogenicity data, which adheres to the carcinogenic classification criteria defined by the IARC [57]. Accordingly, data corresponding to IARC Group 1, 2A, and 2B, which indicate a potential carcinogenic risk, were labeled as ‘carcinogenic positive’. Conversely, data corresponding to Group 3 or showing no carcinogenic evidence were labeled ‘carcinogenic negative’ (Table 2).

**Table 2:** Carcinogenicity labeling of T3DB data based on IARC classification.

**IARC classification**	**Descriptions**	**Label**
Group 1	Carcinogenic to humans	Positive
Group 2A	Probably carcinogenic to humans	Positive
Group 2B	Possibly carcinogenic to humans	Positive
Group 3	Unclassifiable carcinogenic status to humans	Negative

Records from CEBS and IARC were labeled as ‘carcinogenic positive’, as these datasets exclusively contain carcinogenic-positive entries and therefore required no further labeling. For ICE and ToxRefDB, records filtered through *in vivo* study data were labeled as ‘carcinogenic positive’ when experimental conditions in which carcinogenic responses were observed upon compound administration were available.

### Data integration and preprocessing

To integrate the collected databases, any data with unclear or redundant tissue names (e.g. upper digestive tract, respiratory organs, etc.) were removed, and mixtures of multiple compounds, single elements, and ionic compounds that cannot form graph structures were also excluded. Then, compounds with one or more toxic dose 50% (TD50) values or experimentally confirmed as carcinogenic were labeled carcinogenic [20, 58]. To construct organ-level datasets, we grouped the data into organ categories based on tissue, carcinoma, and tumor type information. The detailed mapping information is provided in Supplementary data: Table S1 available online at http://bib.oxfordjournals.org/. We chose four organs in which compounds can cause cancer and that represent the most frequent cancers in the statistics of the Global Cancer Observatory: liver cancer, lung cancer, stomach cancer, and breast cancer [59]. CPDB, IRIS, T3DB, CEBS, IARC, ICE, and ToxRefDB were used for training and validation, whereas the CCRIS was reserved for testing. To avoid data leakage, compounds overlapping with the training and validation sets were removed before constructing the test set. For the negative labeling in the training and validation dataset, we used explicit non-carcinogenic annotations when available. In cases where no explicit non-carcinogenic data were provided, negative labels were implicitly assigned based on organ-specific tumor reporting. Specifically, if a compound was reported to induce tumors in one of the four organs, it was assumed to be non-carcinogenic in the remaining organs unless explicit evidence to the contrary was available. (Supplementary data: Table S2 available online at http://bib.oxfordjournals.org/). A training and validation dataset comprising 335 compounds was constructed, with each compound annotated with binary carcinogenicity labels across four distinct organ types. Separate test datasets were prepared for each organ type, containing 311, 211, 36, and 89 compounds for the liver, lung, stomach, and breast, respectively (Table 3, Table 4, Supplementary data: Fig. S1 available online at http://bib.oxfordjournals.org/).

**Table 3:** The number of organ-specific positive and negative samples in the carcinogenicity database.

	**Liver**	**Lung**	**Stomach**	**Breast**
Positive	176	164	162	163
Negative	159	171	173	172
Total	335	335	335	335

**Table 4:** The number of organ specific positive and negative samples in the test set.

	**Liver**	**Lung**	**Stomach**	**Breast**
Positive	293	161	25	58
Negative	18	50	11	31
Total	311	211	36	89

### Molecular graph feature vector generation

**Figure 1:**
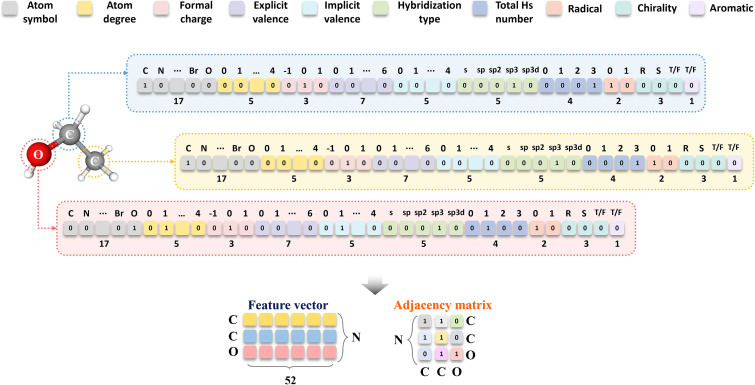
Example of a created molecular feature vector and adjacency matrix using ethanol. A 52-dimensional feature vector was generated for each non-hydrogen atom in the molecule, including two carbons and one oxygen. The generated feature vectors were combined into a single vector of size 52 × *N*, where *N* is the number of atoms. Additionally, an adjacency matrix was created to represent the connectivity information where a value of 1 indicates that two atoms are connected, and a value of 0 indicates that they are not connected.

We used the RDKit library to extract the structure and chemical properties of compounds from SMILES information, which is a string representation of a molecular structure [60]. The RDKit library provides functions for molecular structure analysis, which extracts atom and bond information from the SMILES string. The extracted data are converted into a graph structure with each atom in the molecule as a node and the bonds between atoms as edges. Each atom node is represented by a 52-dimensional feature vector based on 10 properties, which reflects the chemical and structural information (Fig. 1). These properties include the atom symbol, atom degree, formal charge, explicit valence, implicit valence, hybridization type, the total number of bonded hydrogen atoms, radicals, chirality, and aromaticity. A description is provided for each of the 10 atom properties, example values, and the number of encoding dimensions (Table 5). Additionally, the adjacency matrix is a two-dimensional array of connections between atoms and atoms in a graph. The adjacency matrix is constructed as a matrix of size $N\times N$, where $N$ is the number of nodes, and provides information about the connections between nodes.

**Table 5:** Atom feature vectors and dimensions for molecular modeling.

**Atom representation**	**Descriptions**	**Features**	**Dimensions**
Atom symbol	The symbol of the element represented using one hot encoding	C, N, Br, O…	17
Atom degree	The number of neighboring atoms directly bonded to the atom	0, 1, 2, 3, 4	5
Formal charge	The formal charge value of the atom	-1, 0, 1	3
Explicit valence	The number of electrons the atom has available for forming bonds with other atoms	0, 1, 2, 3, 4, 5, 6	7
Implicit valence	The number of additional bonds the atom can potentially form	0, 1, 2, 3, 4	5
Hybridization type	The hybridized orbital state of the atom	s, sp, sp2, sp3, sp3d	5
Total Hs number	The total number of hydrogen atoms bonded to the atom	0,1,2,3	4
Radical	The number of radical electrons the atom possesses	0,1	2
Chirality	Whether the atom is a chiral center and the arrangement of its substituents around that center	R, S, T/F	3
Aromatic	Whether the atom has aromatic characteristics	T/F	1
Total	52		

### Model overview

**Figure 2:**
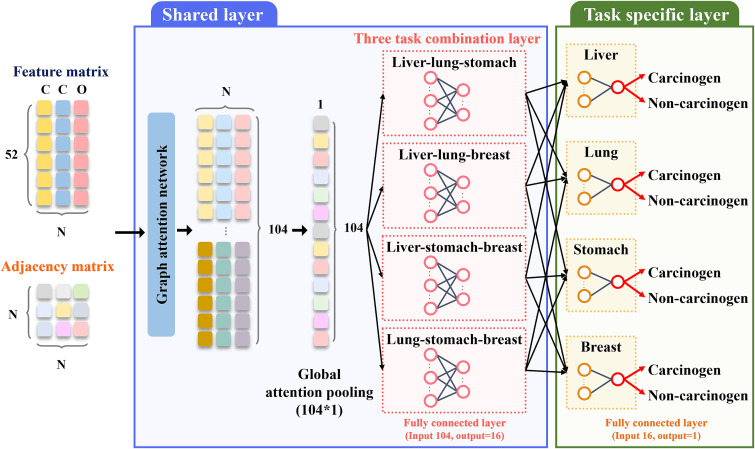
Graph-based multi-task model structure for compound carcinogenicity prediction. The adjacency matrix and feature vectors of the compounds are used as input for the model and passed through the GAT layer of the shared layer. After multi-head attention with two heads, the feature vectors are transformed to 104 × N and reduced to 104 × 1 vector by global attention pooling. The vector is input to the three task combination layer corresponding to the organ information related to the carcinogenicity of the compound, e.g. if a compound has carcinogenicity information about the liver, lung, and stomach, it is input to that layer. This information is passed through the task-specific layers to generate organ-specific carcinogenicity predictions.

We proposed a GAT-based multi-task model capable of learning cross-organ representations and performing various organ-specific tasks. The input for the model is provided in the form of molecular graphs, where a 52-dimensional feature vector represents each atom. This feature vector is processed through the GAT layer, where the features of each atom are updated through interactions with its neighbors. The GAT is applied in the first layer using the attention mechanism to learn how important each neighboring node is to the target node. Next, global attention pooling is applied after updating the features of each node through the GAT layer. This pooling mechanism learns weights for the nodes and aggregates their features to produce a 104 × 1 vector representing the entire graph [61]. The model is trained using three-task combinations to predict the four tasks (liver, lung, stomach, and breast). Independent, fully connected layers are constructed for each task combination, and the pooling vector is passed through these layers. The vector provided to each layer reflects to the compound’s carcinogenicity profile with respect to multiple organs. After passing through the layers for each task combination, the vectors pass through the task-specific layer to generate the carcinogenicity prediction for each task (Fig. 2).

### Training multi-task model

**Figure 3:**
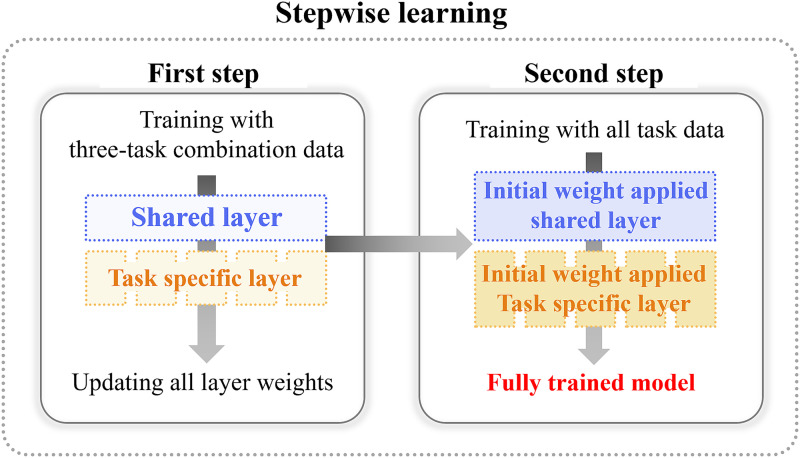
The stepwise learning process for the proposed model. In the first step, the model weights are updated using only the data from three-task combinations. The updated weights are then used as the initial weights for the second step. In the first step, the layers are shown before their weights have been updated, while in the second step the same layers are shown with the learned weights applied.

The proposed stepwise multi-task learning consists of two steps, where each step is designed to maximize the interaction between tasks during the learning process. In the first step of learning, three of the four organ carcinogenicity prediction tasks are combined, resulting in four combinations: liver–lung–stomach, liver–lung–breast, liver–stomach–breast, and lung–stomach–breast. To train each combination, we used a corresponding dataset that contains only the binary carcinogenicity labels for the selected three organs (Table 6, Supplementary data: Table S3 available online at http://bib.oxfordjournals.org/). The datasets used in this step are entirely separate from the organ-specific dataset (see Table 3); they do not overlap and are relatively smaller in size. These datasets were used to support task-aware initialization by capturing shared patterns among related tasks before training started on the organ-specific dataset. Each combination layer operates independently, focusing on learning features specific to its task combination. This step allows the model to learn important features shared in the three-task combinations while obtaining the optimal weights for each combination to be used as initial weights in the next step. In the second step, the model is trained using the initial weights learned in the first step. Using these initial weights enables faster and more reliable learning when the model is finally trained on data containing labels for all four tasks. Through this stepwise learning process, the model sufficiently reflects the task interactions in the first step from three-task combinations and then in the second step with the entire dataset (Fig. 3).

**Table 6:** Three-organ combinations carcinogenicity database for the first step learning.

	**The number of three organ combination data**
Liver–lung–stomach	89
Liver–lung–breast	63
Liver–stomach–breast	30
Lung–stomach–breast	34
Total	216

Hyperparameters such as learning rate, weight decay, and dropout rate were optimized through grid searches (see Supplementary data: Tables S4, and S5 available online at http://bib.oxfordjournals.org/) [62]. To identify the optimal hyperparameter configurations, stratified 10-fold cross-validation was applied to the organ-specific dataset (see Table 3), ensuring that each fold preserved both the organ-specific data distribution and the overall label balance between carcinogens and non-carcinogens [63]. Using the optimized hyperparameters, the model was then trained on a designated training set with early stopping based on validation performance. Finally, an independent external dataset was used as a hold-out test set for final evaluation (Supplementary Fig. S2). Additionally, to address the issue of sample imbalance within the dataset, the focal loss was used as the loss function [64]. Focal loss is a variation of binary cross entropy that applies additional weighting for class imbalance to help the model focus more on difficult predictions. Focal loss is defined as shown in Formula 1, where ${P}_t$ represents the probability of the class predicted by the model, ${\alpha}_t$ denotes the class-specific weight, and $\gamma$ is the focusing parameter, which is used to assign greater weight to difficult examples. We adopted the values recommended by the original focal loss paper (${\alpha}_t$=0.25, $\gamma$=2), which were reported to yield the best performance, to reduce the imbalance between carcinogenic and non-carcinogenic samples.


(1) \begin{equation*} FL\left({P}_t\right)=-{\alpha}_t{\left(1-{p}_t\right)}^{\gamma}\mathit{\log}\left({p}_t\right) \end{equation*}

To accurately measure prediction probabilities for the binary classification tasks, task-specific loss was calculated and then summed across tasks to compute the overall loss. Through this process, the parameters of the model were updated to minimize the loss by considering the losses from all tasks.

### Calculating attention weights

The global attention pooling method generates a single representative feature vector for the entire graph. This method computes and normalizes a learnable importance score for each node embedding. The graph representation vector is then produced as a weighted sum of the transformed features. This approach makes it possible to identify important nodes in the overall graph, as shown in Formula 2. In this formula, ${r}_i$ represents the global attention pooling vector in the graph $i$, where ${N}_i$ is the number of nodes in the graph, and ${x}_n$ is the node feature vector for the node $n$. The function ${h}_{gate}\left({x}_n\right)$ implemented as a simple multi-layer perceptron computes the importance of each node that produces a scalar. These scalars are normalized by $softmax$ to yield attention weights ${a}_n$, which sum to one. Attention weights ${a}_n$ reflects how the critical node $n$ is to the overall representation. The function ${h}_{\Theta}\left(\bullet \right)$ then transforms the features of each node ${x}_n$ producing ${h}_{\Theta}\left({x}_n\right)$. By multiplying the ${a}_n$ with the transformed features ${h}_{\Theta}\left({x}_n\right)$, the importance of each node is reflected in its feature representation. Finally, the information from all nodes is aggregated by summing the weighted feature representations to produce the global representation ${r}_i$of graph $i$. Instead of mean pooling or max pooling, this approach provides a pooling mechanism using learnable importance to generate a graph representation.


$$ attention\ weights={a}_n= softmax\left({h}_{gate}\left({x}_n\right)\right) $$

(2) \begin{equation*} {r}_i=\sum_{n=1}^{N_i}{a}_n\bigodot{h}_{\Theta}\left({x}_n\right) \end{equation*}

### Model evaluation metrics

The evaluation of this model was conducted for each task using performance metrics such as the area under the receiver operating characteristic curve (AUROC) and the area under the precision–recall curve (AUPR). These allow for evaluating how effectively the model generalizes for each task. Formula (3) was used to calculate the performance metrics, where TP, FP, TN, and FN represent true positives, false positives, true negatives, and false negatives, respectively.


$$ TPR= Recall=\frac{TP}{TP+ FN} $$

$$ FPR=\frac{FP}{FP+ TN} $$

(3) \begin{equation*} Precision=\frac{TP}{TP+ FP} \end{equation*}

The AUROC is a metric that represents the probability of the model, correctly classifying positive classes as positive and negative classes as negative, and it is calculated using the true positive rate (TPR) and false positive rate (FPR). The TPR is the percentage of true positives that are correctly predicted to be positive, and the FPR is the percentage of true negatives that are incorrectly predicted to be positive. The ROC curve is a graph that presents the FPR on the *x*-axis and the TPR on the *y*-axis, and the AUROC is the area under this curve. The AUROC value ranges from 0 to 1, with values closer to 1 indicating better discrimination between positive and negative classes. The AUPR is a useful metric for evaluating model performance on imbalanced datasets, calculated using precision and recall. Precision refers to the proportion of true positives among the samples predicted as positive, while recall is equivalent to the TPR and represents the proportion of actual positives correctly predicted. The PR curve is a graph with recall presented on the *x*-axis and precision on the *y*-axis; the AUPR represents the area under this curve. The AUPR value ranges from 0 to 1, with values closer to 1 indicating that the model effectively predicts positive samples even when there is a larger amount of negative data.

## Results

### Performance evaluation of carcinogenicity prediction

We compared the evaluation results of our multi-task, single-task, and the current best-performing carcinogenicity prediction single-task models, Mol-BERT [21], CarcGC [30], DCAMCP [37], XGBoost, support vector machine (SVM), and random forest, to assess their performances (Table 7). The architecture of our single-task model is displayed in Supplementary Fig. S3. Except for our multi-task model, all other models were trained as organ-specific single-task models, each designed to predict carcinogenicity in a specific organ (liver, lung, stomach, or breast). For Mol-BERT, fine-tuning was performed on our training data prior to evaluation on the test set. Our multi-task model consistently achieved superior AUROC and AUPR across the liver, lung, and stomach organs, demonstrating clear advantages over all competing approaches. Notably, the highest AUROC was recorded in the stomach task (0.7636), outperforming all comparative models (0.5527–0.7418). The highest AUPR was observed in the liver task (0.9646), surpassing all comparative models (0.9373–0.9621). These gains highlight the key advantage of the multi-task learning framework. By jointly training across multiple organ types, the model is able to leverage shared molecular features and cross-organ carcinogenicity patterns that organ-specific single-task models inherently cannot capture. In the breast organ, however, our model achieved lower performance than Mol-BERT (AUROC 0.7508; AUPR 0.8385) and Random Forest (AUROC 0.7241; AUPR 0.8174), suggesting that breast carcinogenicity may be driven by more organ-specific molecular mechanisms that are less amenable to shared representation learning.

**Table 7:** Performance evaluation of multi-task, single task, Mol-BERT, CarcGC, DCAMCP, XGBoost, and random forest models.

**Organ**	**Model**	**AUROC**	**AUPR**
**Liver**	Multi-task (Ours)	**0.7023**	**0.9646**
	Single-task	0.5576	0.9505
	Mol-BERT	0.6613	0.9566
	CarcGC	0.6196	0.9495
	DCAMCP	0.5502	0.9373
	XGBoost	0.5651	0.9554
	Random forest	0.6211	0.9621
**Lung**	Multi-task (Ours)	**0.7486**	**0.8907**
	Single-task	0.6789	0.8255
	Mol-BERT	0.7146	0.8744
	CarcGC	0.7301	0.8685
	DCAMCP	0.6384	0.8377
	XGBoost	0.6272	0.8562
	Random forest	0.6870	0.8691
**Stomach**	Multi-task (Ours)	**0.7636**	**0.8914**
	Single-task	0.7055	0.8085
	Mol-BERT	0.7091	0.8844
	CarcGC	0.7418	0.8846
	DCAMCP	0.5527	0.6881
	XGBoost	0.6872	0.8652
	Random forest	0.6709	0.8338
**Breast**	Multi-task (Ours)	0.6813	0.7408
	Single-task	0.6240	0.7613
	Mol-BERT	**0.7508**	**0.8385**
	CarcGC	0.5512	0.7316
	DCAMCP	0.5895	0.7206
	XGBoost	0.5874	0.7330
	Random forest	0.7241	0.8174

In addition, to investigate which chemical classes are well- and poorly predicted by the model, we performed a chemical class–stratified analysis using SMARTS (SMiles ARbitrary Target Specification) based annotation across 42 structural classes; the full results are provided in the Supplementary data (Figs S4 and S5, Table S6 available online at http://bib.oxfordjournals.org/).

### Analysis of gradient cosine similarity

**Figure 4:**
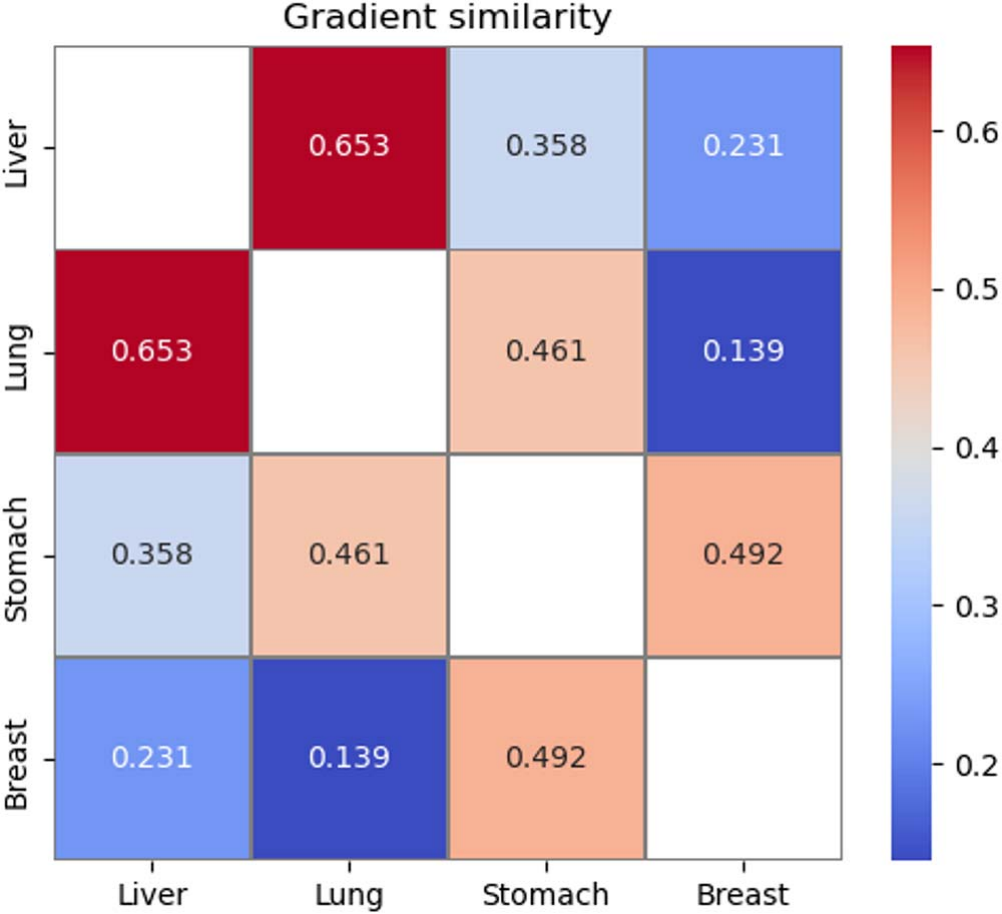
Gradient cosine similarity observed through the heatmap. Task-specific gradients were extracted from the shared layer, and pairwise gradient cosine similarity analysis was conducted. The heatmap reveals that the similarity between liver and lung is the highest, whereas the similarity between lung and breast is the lowest.

To explain the results of the performance evaluation, we examined the gradient cosine similarity between task pairs of the shared layer parameters (Fig. 4). A higher gradient cosine similarity indicates that the directions in which tasks update the shared layer parameters are similar [65]. A comparison of the cosine similarity of shared layer gradients collected during the second step learning revealed distinct patterns of task relationships. The liver–lung pair exhibited the highest gradient similarity (s_grad_), suggesting that these two tasks share highly compatible feature representations in the shared layers. Notably, the stomach task maintained moderate similarity with all three other tasks (s_grad_ = 0.358–0.492) without extreme conflict with any particular task, which may have contributed to its stable learning. In contrast, the breast task showed the lowest gradient similarity with both lung (s_grad_ = 0.139) and liver (s_grad_ = 0.231), indicating that its parameter updates were largely independent from those of the other major tasks. This suggests that the features required for the breast task differed from those of the other tasks, limiting the benefits of shared feature learning.

### Substructure analysis of carcinogenic molecules

To analyze the substructures crucial for predicting carcinogenic compounds, we investigated the molecular substructures with high attention weights among the carcinogen compounds that achieved high prediction scores in the test set. The molecular substructures of compounds with high prediction scores were highlighted visually, and evidence-based analysis using existing scientific literature was conducted to confirm whether these substructures are related to carcinogenicity.

**Figure 5:**
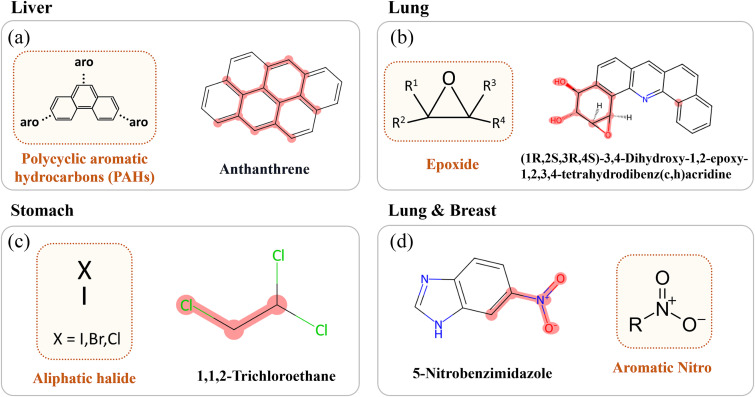
Organ-specific carcinogen substructures with attention weights. (a) Compounds that exhibit carcinogenicity in the liver and (b) in the lung. (c) Compounds that exhibit carcinogenicity in the stomach, (d) in the lung, and breast. The highlighted regions indicate areas in the molecule with high attention weights.

For Anthanthrene, which exhibited carcinogenicity in the liver, the model highlighted the polycyclic aromatic hydrocarbon (PAH) (Fig. 5a). PAHs can be metabolized into reactive species, such as diol epoxides and PAH cations, which covalently bind to purine bases in DNA, forming unstable DNA adducts that can lead to replication errors [66, 67]. Ultimately, these events may result in oncogenic transformations [68–72].

Moreover, the model highlighted the epoxide substructure in (1R,2S,3R,4S)-3,4-Dihydroxy-1,2-epoxy-1,2,3,4-tetrahydrodibenz(c,h)acridine, which showed carcinogenicity in the lung (Fig. 5b). Various epoxides and epoxide-forming chemicals have been proven to be carcinogenic. Epoxides act as strong alkylating agents and can form covalent bonds with DNA, potentially causing mutations and carcinogenesis. Epoxides can also react with biological nucleophiles *in vivo* (e.g. protein residues, DNA/RNA bases), leading to DNA damage [73, 74].

1,1,2-Trichloroethane, which features an aliphatic halide substructure, exhibited carcinogenicity in the stomach (Fig. 5c). Aliphatic halides can be metabolically activated into a reactive intermediate capable of covalent DNA binding, thereby inducing mutagenic and carcinogenic effects [75, 76].

Lastly, the carcinogenic compound 5-Nitrobenzimidazole highlights the aromatic nitro substructure in the lung and breast (Fig. 5d). Aromatic nitro compounds are metabolized sequentially into aromatic nitroso, aromatic hydroxylamines, and anilines [77]. These metabolites of aromatic nitro compounds are known to form covalent bonds with DNA, which can lead to mutations and carcinogenicity [78–80].

## Discussion

Compared to previous methods that treat tasks independently, this proposed multi-task model learns more abundant cross-organ representations by reflecting shared features across task combinations. The multi-task model achieved the best performance in liver, lung, and stomach carcinogenicity prediction. However, for breast carcinogenicity prediction, it showed limited improvement relative to some baselines while still ranking third in AUROC among all compared models. This suggests that the benefit of multi-task learning may depend on inter-task relatedness, which was further supported by gradient cosine similarity analysis showing that the breast task had the lowest similarity with other organs. These results indicate that multi-task learning is a promising approach, though its effectiveness is task-dependent and further investigation is needed to identify the conditions under which it provides the greatest benefit.

Furthermore, this study analyzed the specific substructures of carcinogenic compounds that the model prioritized for prediction using attention weights, which represent the learned importance of each node in constructing the final graph representation. As a result, substructures reflecting different carcinogenic mechanisms, such as PAHs, epoxide, aliphatic halide, and aromatic nitro, were identified as key factors for predicting carcinogenicity. These results suggest that the model captures relevant substructures that contribute to carcinogenic potential.

The model developed in this study demonstrates high predictive performance and explainability in all organs, but there are some limitations and improvements to consider. The first issue is the restricted availability of databases, as most databases only record the general carcinogenicity of a compound. Even when using different databases, numerous duplicate compounds exist, which results in a lack of information on organ-specific carcinogenicity. This limitation in training data may reduce the ability to provide organ-specific predictions for new compounds. A closely related issue concerns the scarcity of explicit organ-specific non-carcinogenic data. Due to the lack of verified negative records for individual organs, compounds reported as carcinogenic in at least one organ were implicitly assigned negative labels for the remaining organs in which no carcinogenic activity was recorded. While this approach was a pragmatic necessity given the data constraints, it carries the risk of introducing false negatives into the training set, potentially biasing the model toward underestimating carcinogenic potential in certain organ-compound combinations. To minimize the impact on evaluation, the test set was constructed exclusively from compounds with explicit experimental annotations, thereby ensuring that performance metrics reflect predictions against verified labels. Nevertheless, the influence of implicit negative labels on learned representations during training cannot be fully excluded, and future efforts should prioritize the expansion of organ-specific carcinogenicity databases to reduce reliance on such assumptions. The second is a limitation in multi-task learning, which is generally considered effective for learning with limited data; however, a previous study has shown that multi-task learning requires a dataset large enough to train the minimum number of features [81]. However, due to the lack of carcinogen datasets, there is not enough data on compounds that cause carcinogenicity by organ, making it difficult to make predictions for additional organs. Therefore, we plan to conduct further research once a sufficient dataset is built, as the continued accumulation of experimental data is expected to enable more accurate and detailed organ-specific carcinogenicity analyses. The third limitation is related to the interpretation of the predictions by the model using attention-based substructure analysis. The highlighted substructures reflect learned structural patterns from the training data rather than direct biological relevance to carcinogenicity. Therefore, substructure highlighting based on attention weights should not be interpreted as direct evidence of causality in carcinogenicity, but rather regarded cautiously as potential clues indicating relevance. The last limitation is that the model proposed in this study does not consider factors important for carcinogenicity and toxicity assessment, such as route of administration, dose, and species specificity of compounds. This is due to the lack of structured databases and differences in toxicity evaluation criteria across databases. Therefore, future studies may need to establish structured databases or standardize those existing ones.

## Conclusion

The continuous increase in cancer patients due to exposure to various carcinogenic compounds has highlighted the need for carcinogenicity prediction research. The multi-task learning model proposed in this study improved performance by integrating cross-organ carcinogenic representations across task combinations. This enables more accurate organ-specific carcinogenicity predictions by effectively learning molecular structures and interactions. Moreover, analyzing the substructures of carcinogenic molecules allowed the identification of organ-specific carcinogenic mechanisms and carcinogenic patterns that could be common to multiple organs. These findings may contribute to identifying carcinogenic patterns of concern in populations exposed to carcinogens and provide data for more accurate carcinogenicity predictions. In conclusion, this study can be an important tool to reduce time and costs in evaluating and regulating carcinogenic substances, and it is expected to significantly contribute to developing personalized medicine research and cancer prevention strategies.

**Key Points**

- A multi-task learning framework is proposed to predict organ-specific carcinogenicity in the liver, lung, stomach, and breast by combining shared and task-specific representations.
- In the shared layer, cross-organ representations are captured to extract generalized carcinogenicity patterns, and the task-specific layers learn organ-specific characteristics.
- Attention-based substructure analysis was applied to identify molecular substructures contributing to carcinogenicity predictions for each organ.
- This framework efficiently evaluates organ-specific carcinogens and advances personalized medicine and cancer prevention.

### Author contributions

Yunju Song (Writing – original draft, Conceptualization, Data curation, Formal analysis, Investigation, Methodology, Software, Validation, Visualization), Hwan Choi (Data curation, Formal analysis, Validation, Visualization) Sunyong Yoo (Conceptualization, Writing – review & editing, Funding acquisition, Project administration, Resources, Supervision).

## Supplementary Material

figS1_bbag296

figS2_bbag296

figS3_bbag296

figS4_bbag296

figS5_bbag296

Supplementary_data_bbag296

## Data Availability

The data underlying this article are available in GitHub repository, at https://github.com/bmil-jnu/OS-Carcinogenicity.
