# Supplementary figures and images for: Carcinogenicity prediction via multi-task learning of cross-organ representations with attention mechanisms
Source: Brief Bioinform. 2026 Jun 4;27(3):bbag296. doi: 10.1093/bib/bbag296 (PMC13273428; doi:10.1093/bib/bbag296)

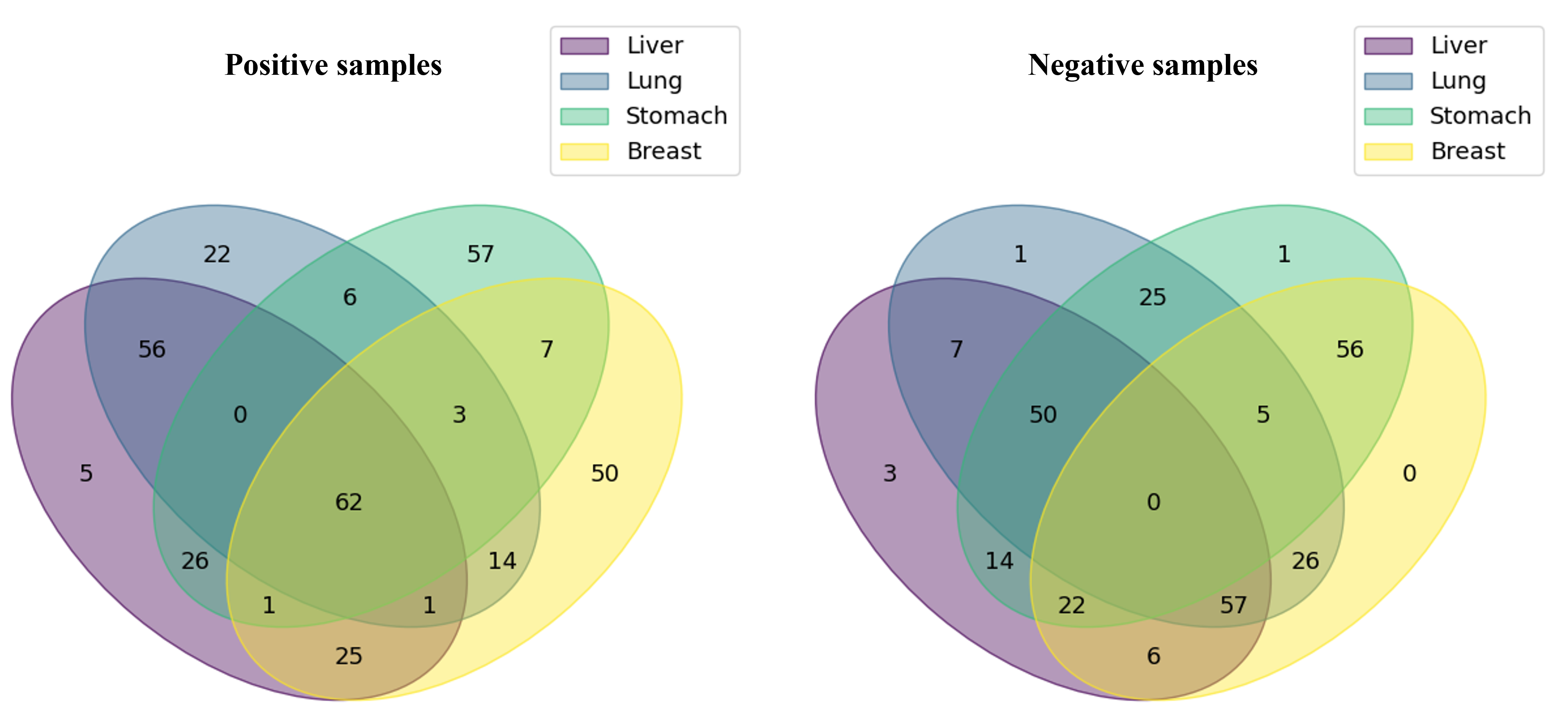

Supplement: figS1_bbag296 [file figS1_bbag296.tif]

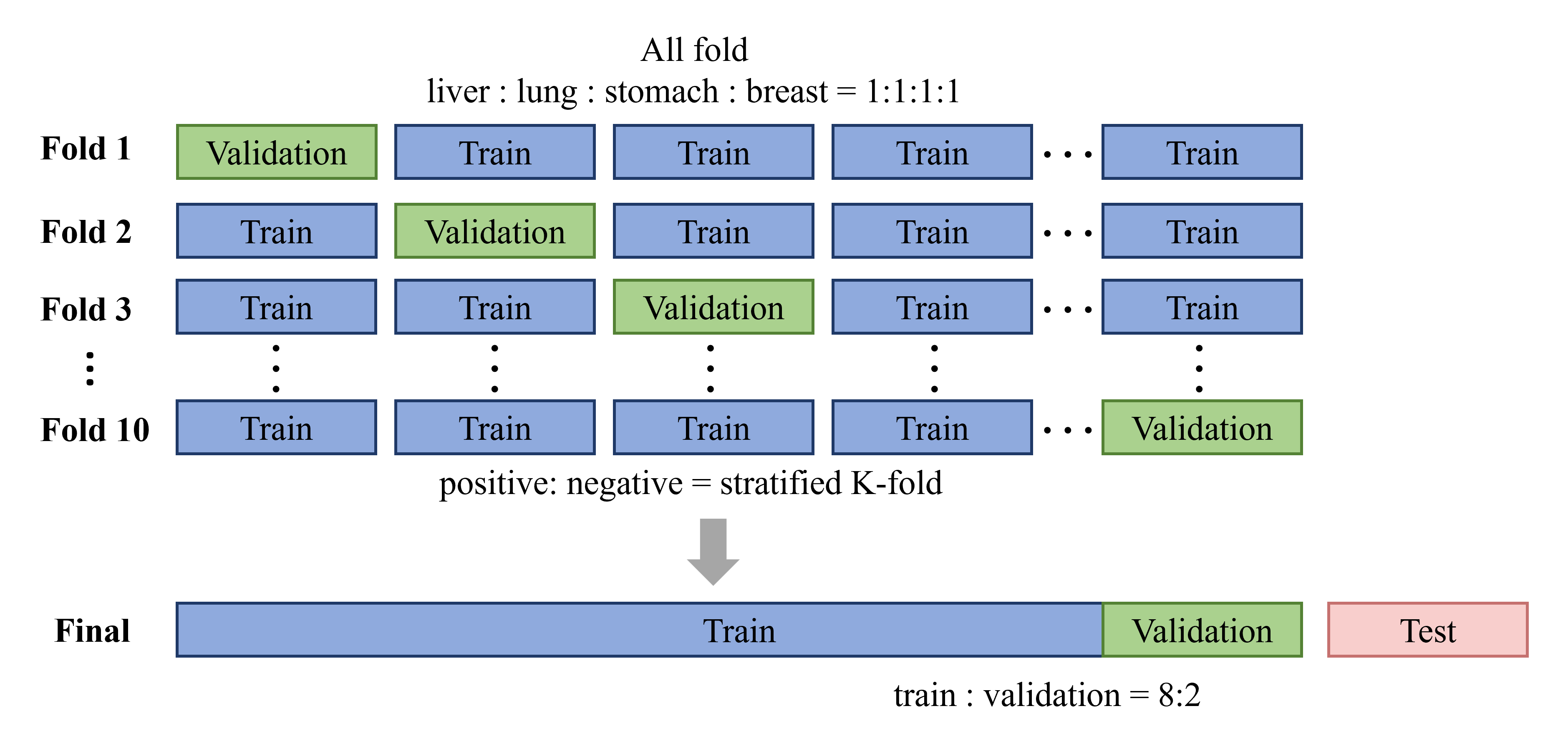

Supplement: figS2_bbag296 [file figS2_bbag296.tif]

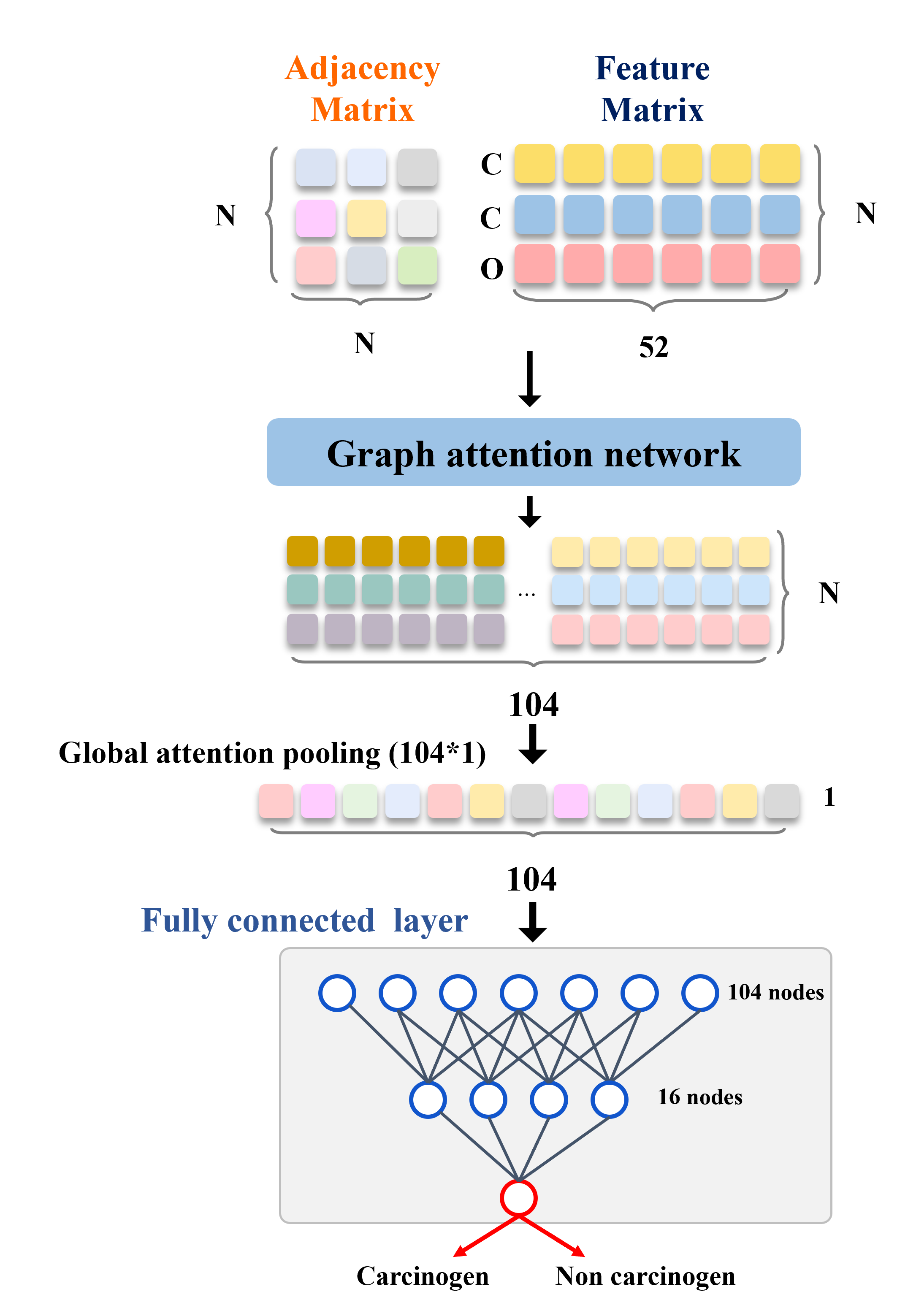

Supplement: figS3_bbag296 [file figS3_bbag296.tif]

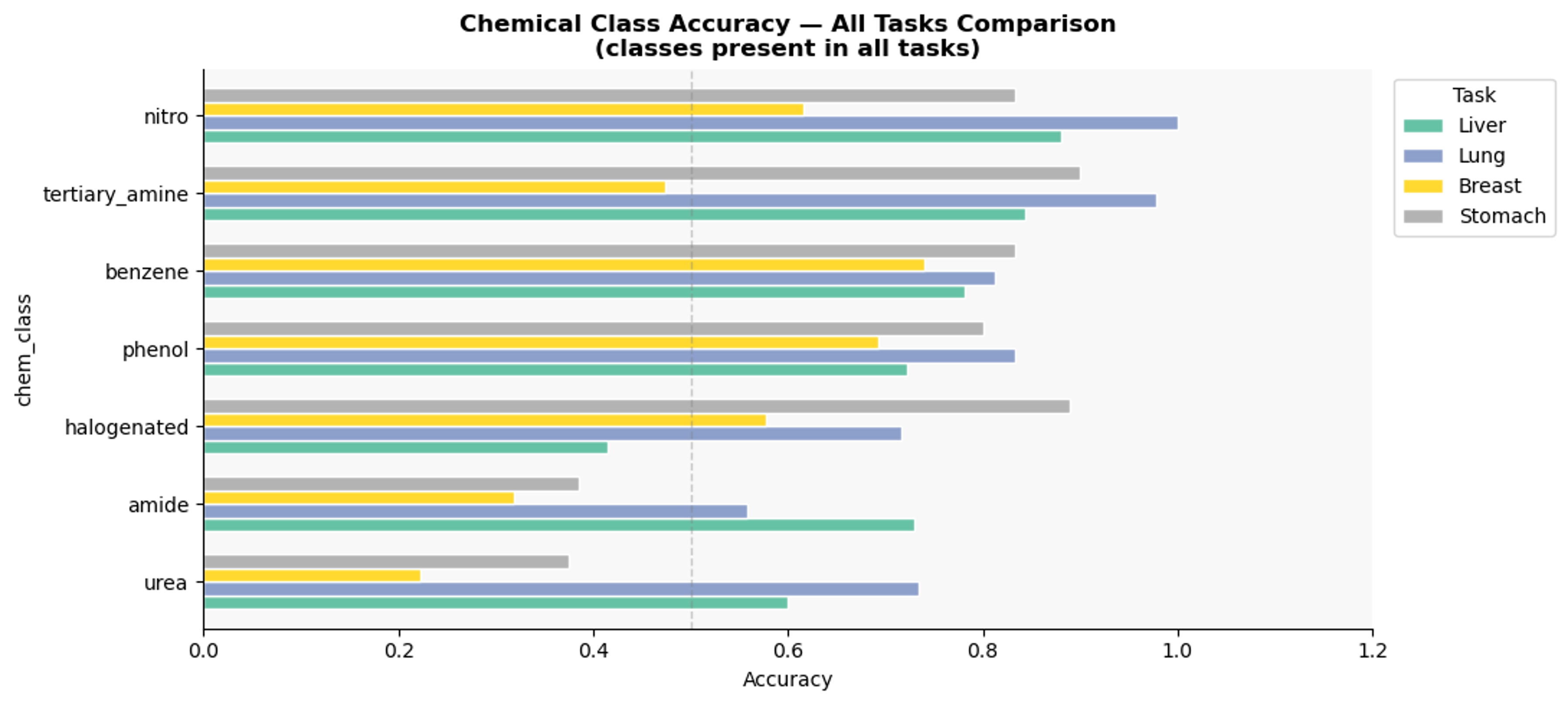

Supplement: figS4_bbag296 [file figS4_bbag296.tif]

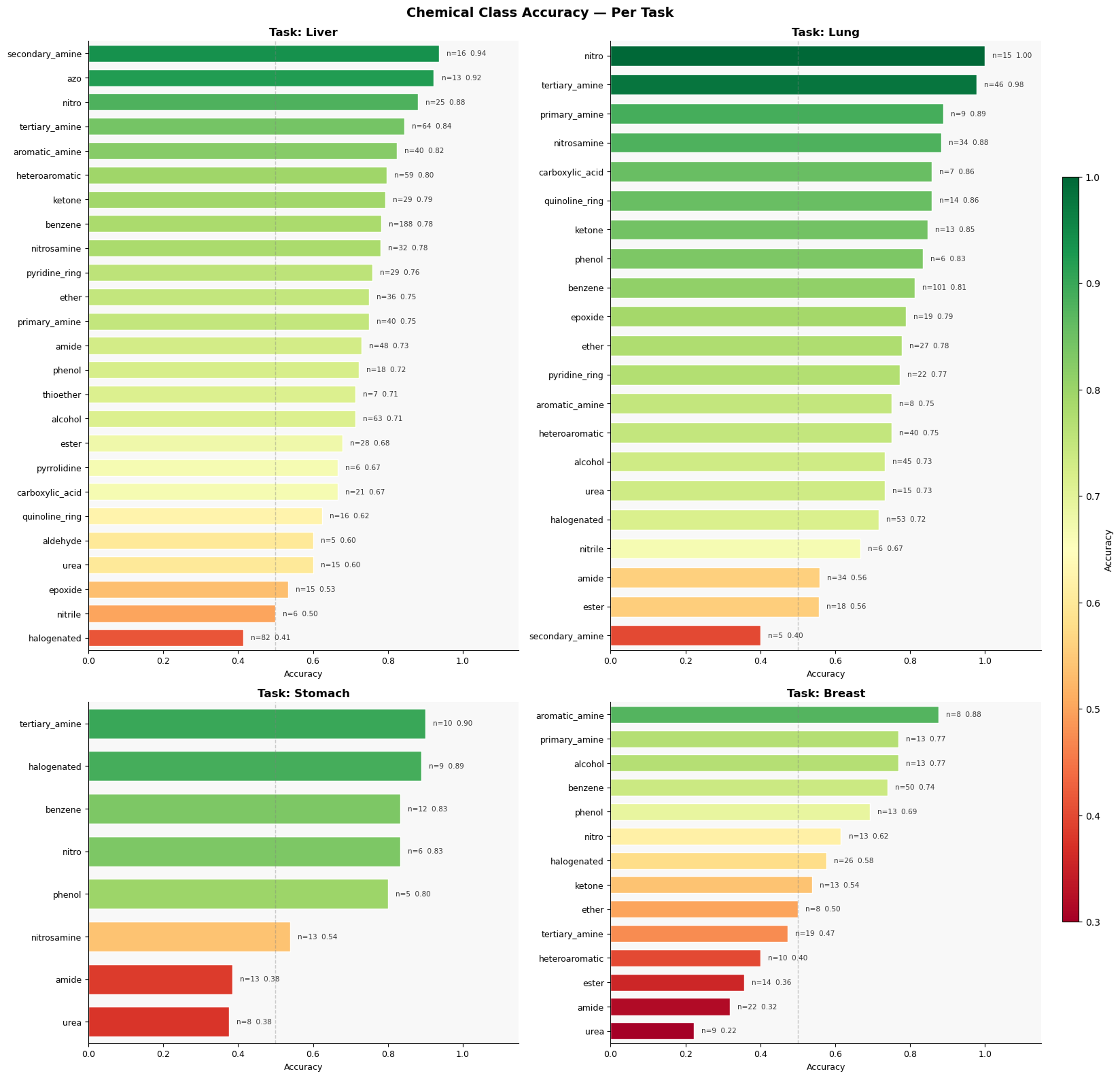

Supplement: figS5_bbag296 [file figS5_bbag296.tif]
