# Supplementary material for: Carcinogenicity prediction via multi-task learning of cross-organ representations with attention mechanisms
Source: Brief Bioinform. 2026 Jun 4;27(3):bbag296. doi: 10.1093/bib/bbag296 (PMC13273428; doi:10.1093/bib/bbag296)
Supplement: Supplementary_data_bbag296 [file Supplementary_data_bbag296.docx]

**Carcinogenicity Prediction via Multi-Task Learning of Cross-Organ Representations with Attention Mechanisms**

**-**

**Supplementary Information**

**Contents**

1. **Supplementary Notes**
   1. Analysis of prediction performance across chemical classes
2. **Supplementary Figure**
3. **Supplementary Table**

**List of Figure**

1. Venn diagram showing carcinogenicity patterns for 335 compounds evaluated across four distinct organ types: liver, lung, stomach, and breast.
2. 10-fold stratified cross-validation scheme designed to achieve balanced task and label distributions.
3. **Structure of the single-task model.**
4. Cross-organ comparison of prediction accuracy for seven common chemical classes.
5. Prediction accuracy by chemical class for each target organ.

**List of Table**

1. List of tumor or carcinoma terminology mapped to each target organ.
2. Distribution of implicit and explicit negative samples in the second step training datasets
3. Class distribution of positive and negative samples in the first-step training datasets.
4. Hyperparameter search details and optimal values for the models
5. Multi-task model architecture and training configuration
6. Classification performance of chemical carcinogenicity prediction by structural class and target organ.
7. **Supplementary Notes**
   1. **Analysis of prediction performance across chemical classes**

To investigate the relationship between chemical functional groups and model performance, we implemented a comprehensive chemical class annotation system using SMARTS (SMiles ARbitrary Target Specification) pattern matching via RDKit. We defined 42 distinct chemical classes organized into three primary categories. The first category comprised 20 common functional groups, including carboxylic acid, ester, amides, urea, sulfonamide, sulfone, amines (primary, secondary, tertiary amine), quaternary ammonium, alcohol, phenol, ether, nitrile, halogenated, heteroaromatic group, aldehyde, ketone, thioether, and benzene ring. The second category included 9 toxicophore alert structures based on established structure-toxicity relationships, encompassing nitro group, nitrosamine, nitroso compound, catechol, quinone, aromatic amine, azo compound, epoxide, and hydrazine. These structural alerts are associated with potential genotoxicity, carcinogenicity, metabolic liability, or high chemical reactivity. The third category consisted of 13 heterocyclic scaffolds commonly found in pharmaceutical compounds, including saturated nitrogen-containing rings (piperidine, piperazine, morpholine, and pyrrolidine), aromatic nitrogen heterocycles (pyridine, pyrimidine, imidazole, indole, benzimidazole, and quinoline), and aromatic oxygen- or sulfur-containing heterocycles (thiophene, furan, and benzofuran). For each of the four organ toxicity prediction tasks, we evaluated how prediction performance varied across different chemical structural classes. In chemical structure analysis, molecules typically contain multiple functional groups simultaneously. For instance, a single molecule might contain an ester group, a tertiary amine, and a pyridine ring, meaning it belongs to three different chemical classes at once. To properly account for the presence of multiple structural features within individual molecules, we designed our analysis so that each molecule could contribute to the performance evaluation of all chemical classes it belonged to. Using the previous example, this molecule's prediction outcome would be counted when calculating performance metrics for the ester class, again for the tertiary amine class, and again for the pyridine ring class. This ensured that we captured the full structural complexity of each molecule rather than forcing an arbitrary choice of which functional group to prioritize.

The analytical workflow proceeded as follows. First, we identified all chemical classes present in each molecule through SMARTS substructure matching. Next, for each organ toxicity task, we grouped all predictions according to their chemical class memberships. Since individual molecules could belong to multiple classes, the same molecule's prediction could appear in multiple chemical class groups. For each chemical class group, we then calculated performance metrics including the number of samples, number of positive and negative cases, accuracy and confusion matrix components (true positives, true negatives, false positives, false negatives). Chemical classes were ranked by prediction accuracy to identify which structural features were associated with superior or inferior model performance. This stratified analysis was conducted independently for each of the four organ toxicity tasks to reveal task-specific relationships between chemical structure and prediction reliability.

To evaluate the predictive reliability of the model across different chemical classes, compounds with n ≥ 5 in the test set were analyzed using per-class accuracy and confusion matrices. Among the chemical classes examined, benzene and phenol demonstrated consistently high predictive accuracy across all four tissues, suggesting that the model captured the structural features of these structure in a tissue-independent manner (Figure S4, Table S6). Within the amine series, tertiary amine maintained accuracy above 0.84 across liver, lung, and stomach, and aromatic amine achieved accuracy above 0.75 in liver, lung, and breast. In contrast, several classes exhibited substantial inter-tissue variability in predictive performance. Halogenated compounds showed the greatest disparity, with accuracy ranging from 0.4146 in liver to 0.8889 in stomach (Δ = 0.4743). Similarly, urea displayed a difference of 0.5111 between lung (0.7333) and breast (0.2222), and tertiary amine showed a gap of 0.5046 between lung (0.9783) and breast (0.4737). Among classes with consistently poor performance across all tasks, amide and urea were the most prominent.

Task-specific analysis further revealed distinct performance patterns across tissues (Figure S5, Table S6). In the liver task, secondary amine, azo, and nitro ranked among the highest-performing classes out of 26 analyzed, whereas halogenated compounds recorded the lowest accuracy at 0.4146. Notably, despite a relatively large sample size (n = 82), halogenated compounds underperformed exclusively in the liver task, implying that this structural class poses particular challenges for liver-specific prediction. Epoxide and nitrile also exhibited poor performance, with accuracy values near 0.5. The lung task showed the most uniform performance among the four tissues, with nitro and tertiary amine achieving accuracy above 0.97, and the majority of classes maintaining accuracy above 0.7, with the exception of nitrile, amide, ester, and secondary amine. The stomach task was limited to nine analyzable classes due to sample size constraints; nevertheless, all classes except nitrosamine, amide, and urea achieved accuracy above 0.80. However, given the small per-class sample sizes (n = 5–13), these results should be interpreted with caution. The breast task exhibited the weakest overall performance distribution among the four tissues, with accuracy falling below 0.5 for urea, amide, ester, heteroaromatic, and tertiary amine. Critically, structural classes that generalized well in other tissues — including tertiary amine and heteroaromatic compounds — failed to do so in breast, strongly indicating that the breast task presents the greatest difficulty for tissue-specific generalization and highlights the inherent complexity of learning tissue-specific carcinogenicity patterns for this organ.

**Supplementary Figure**

**
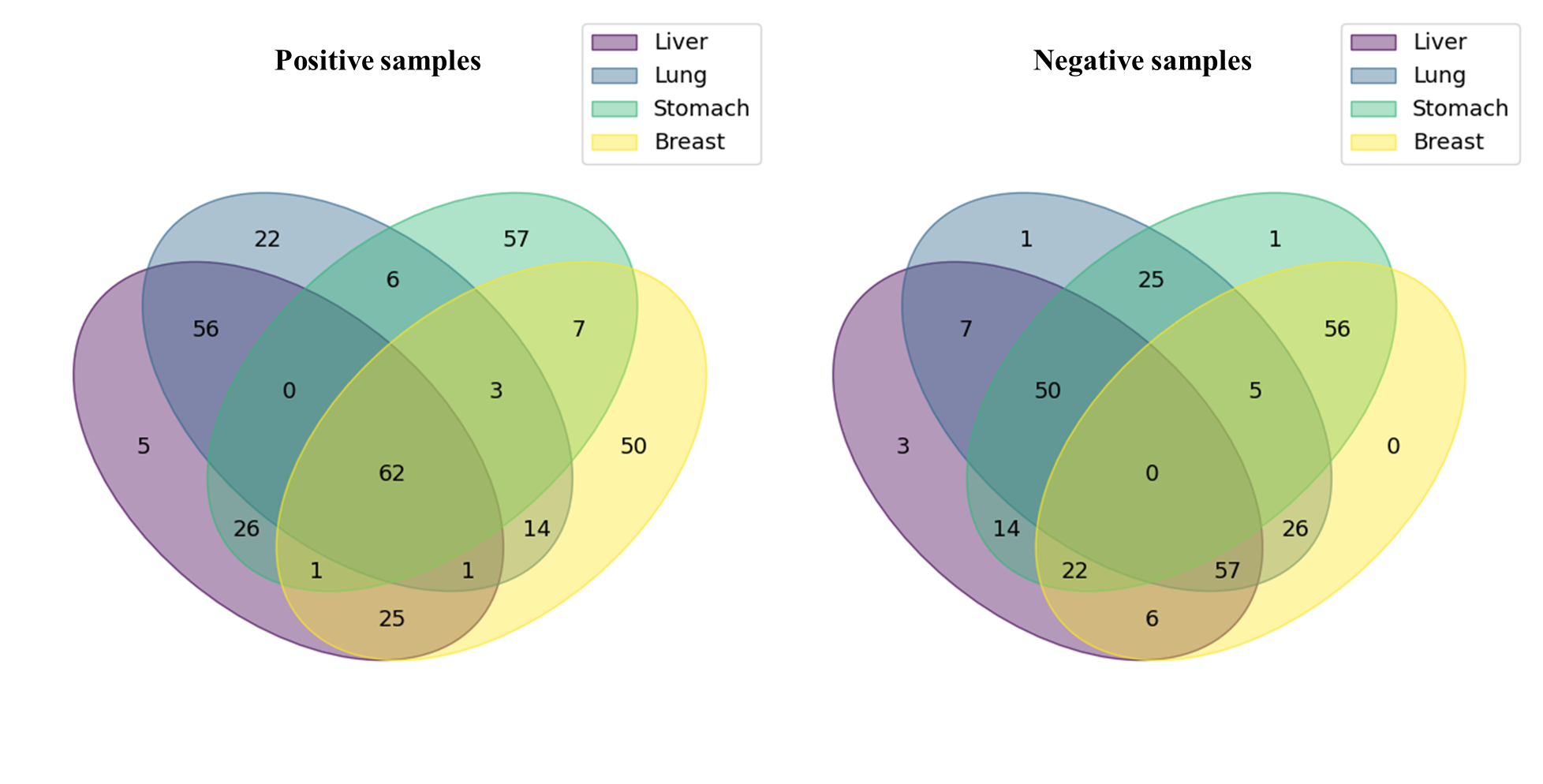
**

**Figure S1** Venn diagram showing carcinogenicity patterns for 335 compounds evaluated across four distinct organ types: liver, lung, stomach, and breast. The left panel shows positive samples, and the right panel shows negative samples. In both panels, each color represents a specific organ: liver (purple), lung (blue), stomach (green), and breast (yellow). Each region of the diagram indicates the number of compounds associated with the corresponding organ combination. In the positive samples panel, the largest group consists of compounds that are carcinogenic across all four organs. At the same time, a substantial number of compounds exhibit carcinogenicity in only a single organ, particularly in stomach, breast, and lung, indicating the presence of organ-specific carcinogenic mechanisms. Many compounds also show carcinogenicity in two or three organ combinations, suggesting shared biological mechanisms across different tissue types. Negative compounds appear across multiple organs due to their definition in the dataset: a compound is labeled negative if no tumors are observed in any tested organ. These distribution patterns support the use of a multi-task learning model that can leverage both shared and organ-specific features.

**
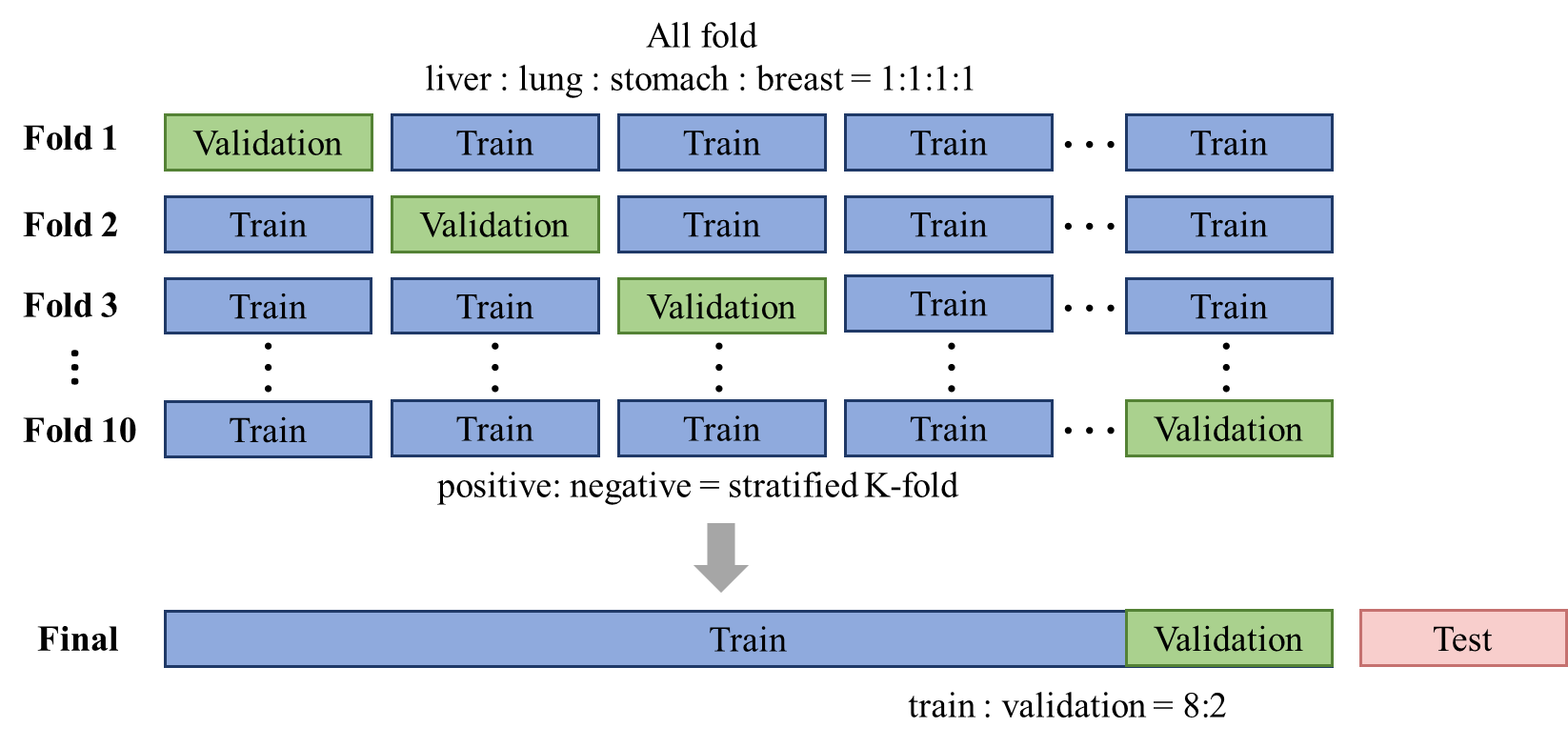
**

**Figure S2** 10-fold stratified cross-validation scheme designed to achieve balanced task and label distributions.

**
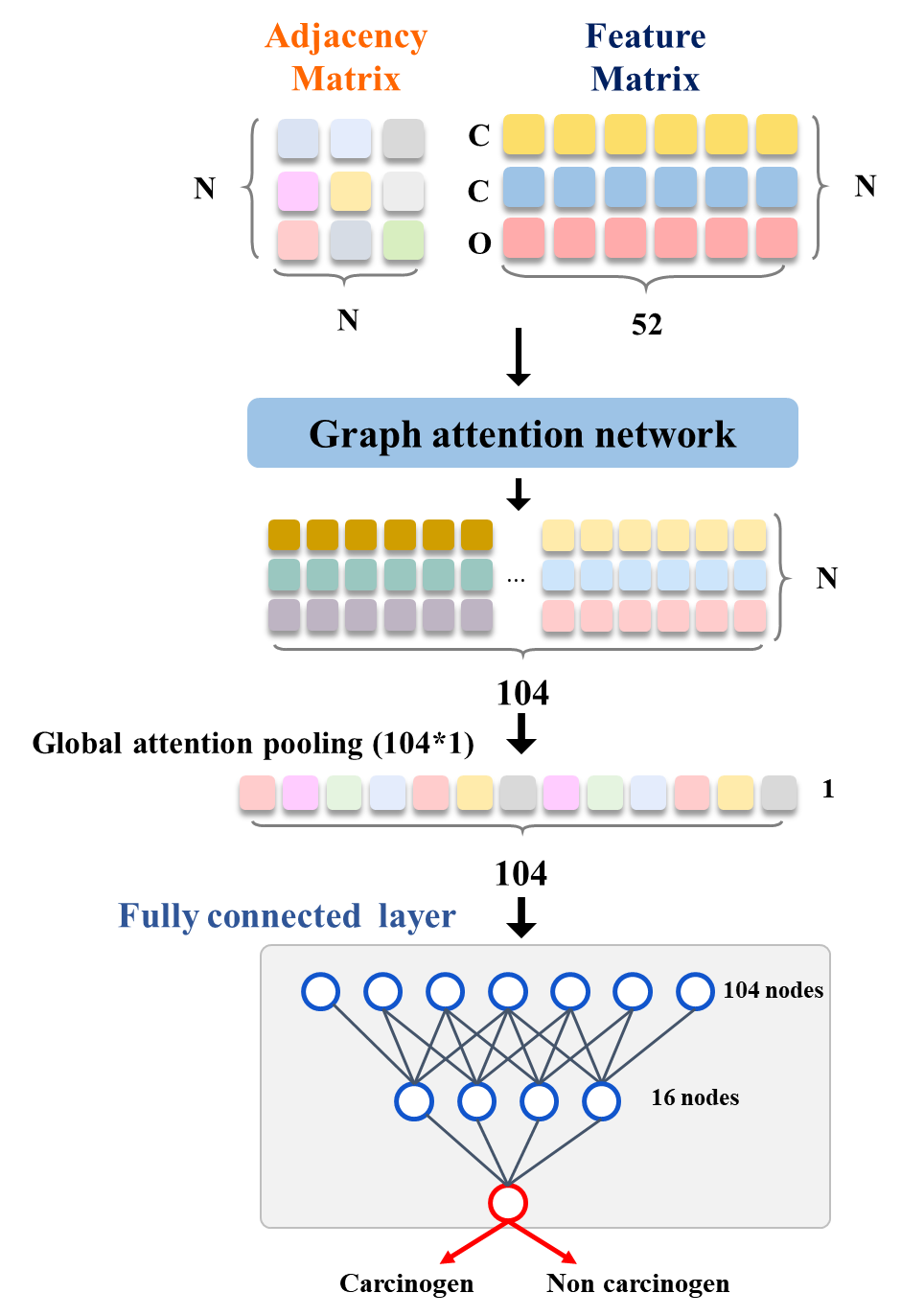
**

**Figure S3** **Structure of the single-task model.** Each single-task model is designed to predict organ-specific carcinogenicity and is trained on data corresponding to a specific organ.

**
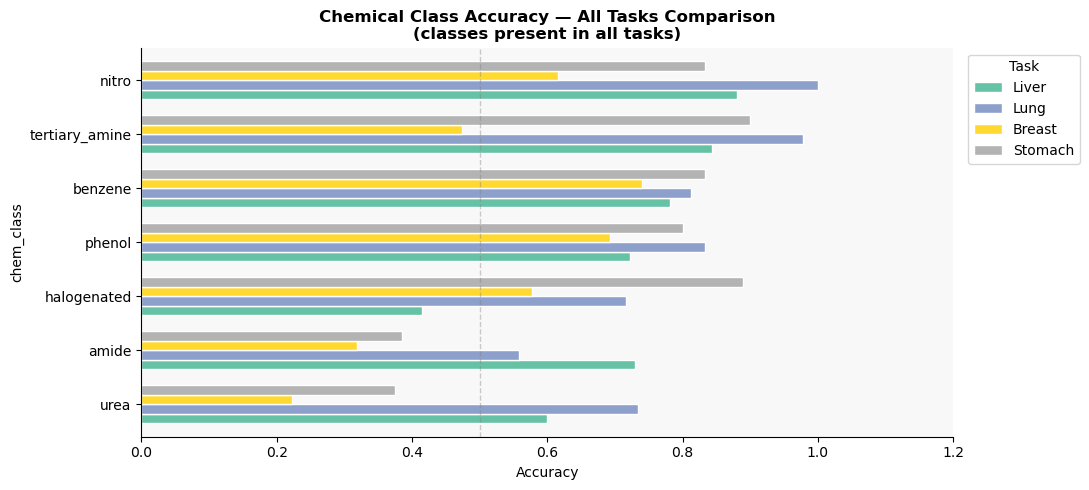
**

**Figure S4** Cross-organ comparison of prediction accuracy for seven common chemical classes.

**
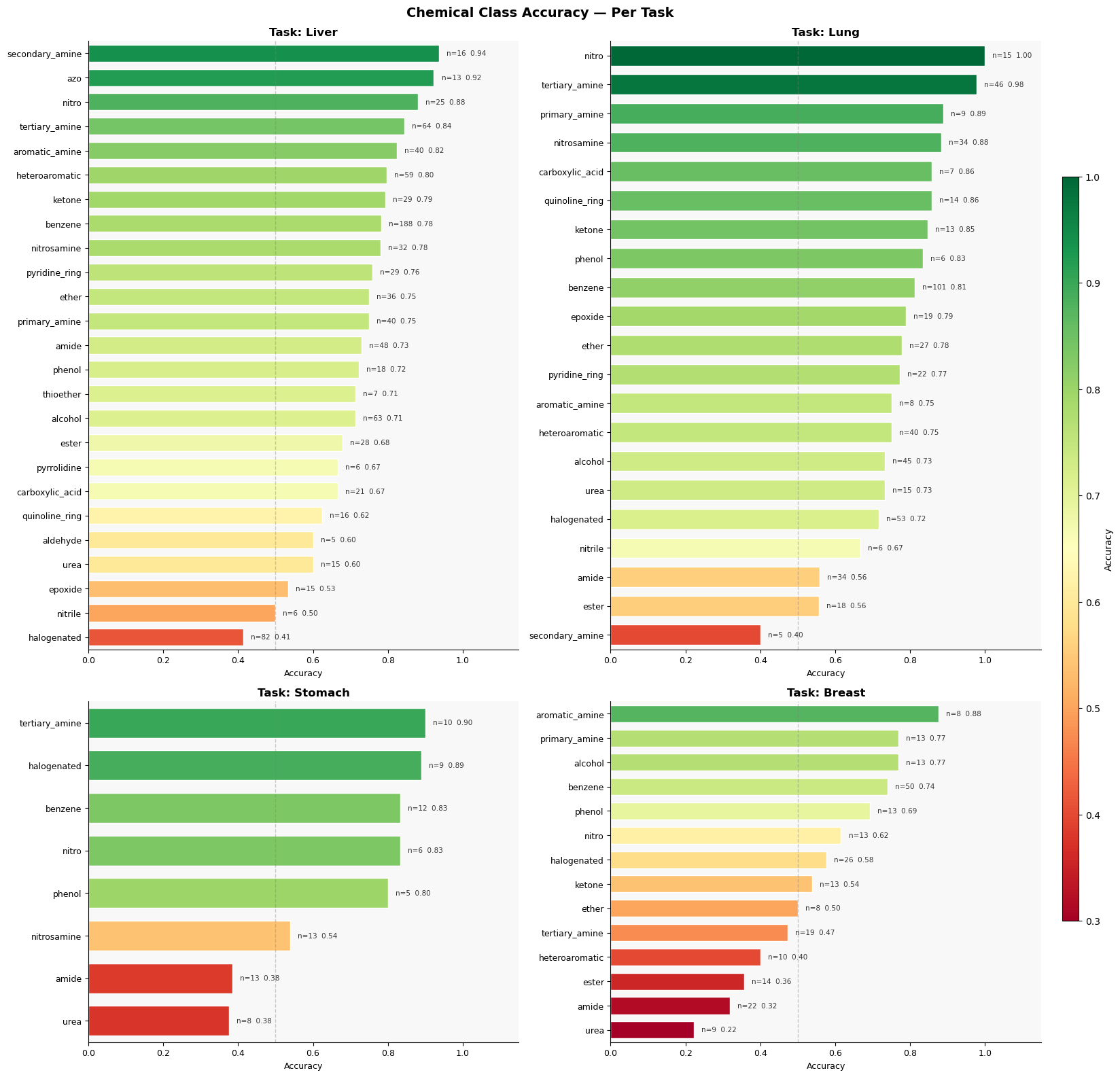
Figure S5** Prediction accuracy by chemical class for each target organ.

**Supplementary Table**

**Table S1** List of tumor or carcinoma terminology mapped to each target organ.

| **Organ** | **Tissue/Carcinoma/Tumor type name** |
| --- | --- |
| **Liver** | Hepatocellular carcinoma |
|  | Hepatocellular carcinomas |
|  | Liver tumors |
|  | Liver carcinoma |
|  | Liver adenoma/ carcinoma combined |
|  | Hepatomas |
|  | Liver angiosarcomas, angiomas, hepatomas, and neoplastic nodules |
|  | Hepatocellular adenoma or carcinoma |
|  | Hepatocellular carcinoma and adenoma |
|  | Hepatocellular adenomas or carcinomas |
|  | Neoplastic liver nodules and carcinomas |
|  | Hepatocellular adenoma/carcinoma |
|  | Hepatocellular carcinomas and neoplastic liver nodules |
|  | Hepatocellular carcinomas and neoplastic nodules |
|  | Liver nodules and hepatocellular carcinomas |
|  | Hepatocellular carcinoma, cholangiocellular carcinoma and adenoma and neoplastic nodules |
|  | Liver hepatocellular adenomas or carcinomas |
|  | Liver hepatocellular adenomas, carcinomas, cholangiomas, or cholangiocarcinomas |
|  | Combined liver nodules and carcinomas |
|  | Hepatocellular adenomas and carcinomas |
|  | Hepatocellular adenoma and carcinoma |
|  | Hepatocellular adenomas or carcinomas and adrenal benign or malignant pheochromocytomas |
|  | Hepatoadenoma and Hepatocarcinoma |
|  | Hepatocellular carcinomas, hepatomas |
|  | Hepatocellular carcinomas or adenomas |
|  | Liver neoplastic nodules or carcinoma (combined) |
|  | Liver adenomas or carcinomas |
|  | Liver tumors, benign and malignant |
|  | Hepatic nodules and hepatocellular carcinomas |
|  | Total of liver angiosarcoma, hepatocellular carcinoma, and neoplastic nodules |
|  | Liver tumors (adenomas and carcinomas; neoplastic nodules and hepatocellular carcinomas) |
|  | liver |
|  | hepatocellular adenoma |
|  | hepatocarcinoma, angiosarcoma |
|  | hepatocellular carcinoma |
|  | hepatocellular adenoma or carcinoma |
|  | hepatoblastoma |
| **Lung** | Lung cancer |
|  | Lung, adenocarcinoma |
|  | Bronchioalveolar adenoma |
|  | Lung cancer and mesothelioma |
|  | Lung, trachea, bronchus cancer deaths |
|  | Cancer mortality from lung cancer and mesothelioma |
|  | Bronchoalveolar carcinomas or adenomas |
|  | lung, bronchus |
|  | lung |
|  | lung, bronchiole |
|  | alveolar/bronchiolar adenoma or carcinoma |
| **Stomach** | stomach |
|  | glandular stomach |
|  | stomach, squamous |
|  | stomach, glandular, fundic region |
|  | stomach, glandular |
|  | stomach, glandular, pyloric region |
|  | gastric mucosa |
|  | stomach, cardiac |
|  | GLANDULAR STOMACH |
|  | pylorus |
|  | stomach, nonglandular |
|  | forestomach, greater curvature |
|  | forestomach, lesser curvature |
|  | forestomach |
|  | Forestomach tumors |
|  | Papillomas and carcinomas of the forestomach |
|  | Forestomach, squamous cell carcinoma |
| **Breast** | mammary |
|  | mammary tissue (other than or including more than mammary gland) |
|  | breast cancer |
|  | mammary gland |
|  | breast |
|  | mammary |

**Table S2** Distribution of implicit and explicit negative samples in the second step training datasets (first-step training datasets contain only explicit data).

|  | **Liver** | **Lung** | **Stomach** | **Breast** |
| --- | --- | --- | --- | --- |
| **Explicit** | 32 | 27 | 34 | 29 |
| **Implicit** | 127 | 144 | 139 | 143 |
| **Total** | 159 | 171 | 173 | 172 |

**Table S3** Class distribution of positive and negative samples in the first-step training datasets.

| **Three-organ combination** | **Total** | **Organ** | **Positive** | **Negative** |
| --- | --- | --- | --- | --- |
| **Liver-Lung-Stomach** | 89 | Liver | 48 | 41 |
|  |  | Lung | 49 | 40 |
|  |  | Stomach | 51 | 38 |
| **Liver-Lung-Breast** | 63 | Liver | 28 | 35 |
|  |  | Lung | 27 | 36 |
|  |  | Breast | 35 | 28 |
| **Liver-Stomach-Breast** | 30 | Liver | 5 | 25 |
|  |  | Stomach | 7 | 23 |
|  |  | Breast | 7 | 23 |
| **Lung-Stomach-Breast** | 34 | Lung | 17 | 17 |
|  |  | Stomach | 17 | 17 |
|  |  | Breast | 16 | 18 |

**Table S4** Hyperparameter search details and optimal values for the models

| **Model** | **Parameters** | **Ranges** | **Optimal values** | | | |
| --- | --- | --- | --- | --- | --- | --- |
|  |  |  | **Liver** | **Lung** | **Stomach** | **Breast** |
| Multi-task (Ours) | Learning rate | [1e-01, 1e-02, 1e-03, 1e-04] | 1e-04 | 1e-04 | 1e-04 | 1e-01 |
|  | Dropout | [0.3, 0.5] | 0.5 | 0.5 | 0.5 | 0.5 |
|  | Weight decay | [1e-03, 1e-04, 1e-05] | 1e-04 | 1e-04 | 1e-04 | 1e-03 |
| Single-task | Learning rate | [1e-03, 1e-04, 1e-05] | 1e-03 | 1e-03 | 1e-03 | 1e-03 |
|  | Dropout | [0.3, 0.5] | 0.3 | 0.3 | 0.3 | 0.3 |
|  | Weight decay | [1e-03, 1e-04, 1e-05] | 1e-05 | 1e-04 | 1e-05 | 1e-05 |
| CarcGC | Learning rate | [1e-03, 1e-04, 1e-05] | 1e-04 | 1e-04 | 1e-04 | 1e-04 |
|  | Dropout | [0.3, 0.5] | 0.3 | 0.5 | 0.5 | 0.3 |
|  | Weight decay | [1e-03, 1e-04, 1e-05] | 1e-03 | 1e-03 | 1e-03 | 1e-04 |
| DCAMCP | Learning rate | [1e-03, 1e-04, 1e-05] | 1e-03 | 1e-03 | 1e-03 | 1e-03 |
|  | Weight decay | [1e-03, 1e-04, 1e-05] | 1e-05 | 1e-04 | 1e-05 | 1e-05 |
| XGBoost | Number of estimators | [100, 200] | 100 | 200 | 200 | 100 |
|  | Maximum depth | [3, 6] | 3 | 6 | 6 | 3 |
|  | Subsample ratio | [0.8, 1.0] | 1.0 | 0.8 | 1.0 | 1.0 |
|  | Column sample by tree | [0.8, 1.0] | 1.0 | 0.8 | 0.8 | 1.0 |
| Random forest | Number of estimators | [50, 100, 150] | 150 | 100 | 100 | 50 |
|  | Maximum depth | [None, 10, 20] | 20 | 20 | None | None |
|  | Minimum samples split | [2, 5] | 5 | 2 | 2 | 5 |
|  | Maximum features | [sqrt, log2] | sqrt | sqrt | log2 | sqrt |

**Table S5** Multi-task model architecture and training configuration

| **Setting** | **Value** |
| --- | --- |
| GAT layers | 1 |
| Attention heads | 2 |
| GAT output dimension | 104 (= 52 × 2) |
| Hidden layer output dimension | 16 |
| Batch size | 2 |
| optimizer | Adam |
| First-step learning epoch (Early stopping) | 205 |
| First-step learning early stopping criteria | 10 |
| Second step learning epoch (Early stopping) | 11 |
| Second step learning early stopping criteria | 10 |

**Table S6** Classification performance of chemical carcinogenicity prediction by structural class and target organ.

| **Task** | **No.** | **Chemical class** | **n** | **pos** | **neg** | **Acc** | **tp** | **tn** | **fp** | **fn** |
| --- | --- | --- | --- | --- | --- | --- | --- | --- | --- | --- |
| **Liver** | 0 | secondary amine | 16 | 15 | 1 | 0.9375 | 14 | 1 | 0 | 1 |
|  | 1 | azo | 13 | 13 | 0 | 0.9231 | 12 | 0 | 0 | 1 |
|  | 2 | nitro | 25 | 22 | 3 | 0.8800 | 19 | 3 | 0 | 3 |
|  | 3 | tertiary amine | 64 | 60 | 4 | 0.8438 | 50 | 4 | 0 | 10 |
|  | 4 | aromatic amine | 40 | 40 | 0 | 0.8250 | 33 | 0 | 0 | 7 |
|  | 5 | heteroaromatic | 59 | 57 | 2 | 0.7966 | 45 | 2 | 0 | 12 |
|  | 6 | ketone | 29 | 29 | 0 | 0.7931 | 23 | 0 | 0 | 6 |
|  | 7 | benzene | 188 | 179 | 9 | 0.7819 | 142 | 5 | 4 | 37 |
|  | 8 | nitrosamine | 32 | 32 | 0 | 0.7813 | 25 | 0 | 0 | 7 |
|  | 9 | pyridine ring | 29 | 28 | 1 | 0.7586 | 21 | 1 | 0 | 7 |
|  | 10 | primary amine | 40 | 39 | 1 | 0.7500 | 29 | 1 | 0 | 10 |
|  | 11 | ether | 36 | 34 | 2 | 0.7500 | 26 | 1 | 1 | 8 |
|  | 12 | amide | 48 | 46 | 2 | 0.7292 | 33 | 2 | 0 | 13 |
|  | 13 | phenol | 18 | 16 | 2 | 0.7222 | 12 | 1 | 1 | 4 |
|  | 14 | thioether | 7 | 7 | 0 | 0.7143 | 5 | 0 | 0 | 2 |
|  | 15 | alcohol | 63 | 61 | 2 | 0.7143 | 43 | 2 | 0 | 18 |
|  | 16 | ester | 28 | 26 | 2 | 0.6786 | 18 | 1 | 1 | 8 |
|  | 17 | pyrrolidine | 6 | 6 | 0 | 0.6667 | 4 | 0 | 0 | 2 |
|  | 18 | carboxylic acid | 21 | 18 | 3 | 0.6667 | 12 | 2 | 1 | 6 |
|  | 19 | quinoline ring | 16 | 15 | 1 | 0.6250 | 9 | 1 | 0 | 6 |
|  | 20 | aldehyde | 5 | 4 | 1 | 0.6000 | 2 | 1 | 0 | 2 |
|  | 21 | urea | 15 | 15 | 0 | 0.6000 | 9 | 0 | 0 | 6 |
|  | 22 | epoxide | 15 | 13 | 2 | 0.5333 | 7 | 1 | 1 | 6 |
|  | 23 | nitrile | 6 | 4 | 2 | 0.5000 | 1 | 2 | 0 | 3 |
|  | 24 | halogenated | 82 | 74 | 8 | 0.4146 | 27 | 7 | 1 | 47 |
| **Lung** | 0 | nitro | 15 | 10 | 5 | 1.0000 | 10 | 5 | 0 | 0 |
|  | 1 | tertiary amine | 46 | 41 | 5 | 0.9783 | 40 | 5 | 0 | 1 |
|  | 2 | primary amine | 9 | 7 | 2 | 0.8889 | 6 | 2 | 0 | 1 |
|  | 3 | nitrosamine | 34 | 34 | 0 | 0.8824 | 30 | 0 | 0 | 4 |
|  | 4 | quinoline ring | 14 | 14 | 0 | 0.8571 | 12 | 0 | 0 | 2 |
|  | 5 | carboxylic acid | 7 | 4 | 3 | 0.8571 | 4 | 2 | 1 | 0 |
|  | 6 | ketone | 13 | 11 | 2 | 0.8462 | 10 | 1 | 1 | 1 |
|  | 7 | phenol | 6 | 3 | 3 | 0.8333 | 3 | 2 | 1 | 0 |
|  | 8 | benzene | 101 | 77 | 24 | 0.8119 | 70 | 12 | 12 | 7 |
|  | 9 | epoxide | 19 | 18 | 1 | 0.7895 | 14 | 1 | 0 | 4 |
|  | 10 | ether | 27 | 24 | 3 | 0.7778 | 20 | 1 | 2 | 4 |
|  | 11 | pyridine ring | 22 | 21 | 1 | 0.7727 | 17 | 0 | 1 | 4 |
|  | 12 | aromatic amine | 8 | 6 | 2 | 0.7500 | 5 | 1 | 1 | 1 |
|  | 13 | heteroaromatic | 40 | 35 | 5 | 0.7500 | 30 | 0 | 5 | 5 |
|  | 14 | alcohol | 45 | 42 | 3 | 0.7333 | 31 | 2 | 1 | 11 |
|  | 15 | urea | 15 | 13 | 2 | 0.7333 | 10 | 1 | 1 | 3 |
|  | 16 | halogenated | 53 | 21 | 32 | 0.7170 | 13 | 25 | 7 | 8 |
|  | 17 | nitrile | 6 | 3 | 3 | 0.6667 | 1 | 3 | 0 | 2 |
|  | 18 | amide | 34 | 26 | 8 | 0.5588 | 17 | 2 | 6 | 9 |
|  | 19 | ester | 18 | 11 | 7 | 0.5556 | 8 | 2 | 5 | 3 |
|  | 20 | secondary amine | 5 | 4 | 1 | 0.4000 | 2 | 0 | 1 | 2 |
| **Stomach** | 0 | tertiary amine | 10 | 7 | 3 | 0.9000 | 6 | 3 | 0 | 1 |
|  | 1 | halogenated | 9 | 3 | 6 | 0.8889 | 2 | 6 | 0 | 1 |
|  | 2 | nitro | 6 | 3 | 3 | 0.8333 | 2 | 3 | 0 | 1 |
|  | 3 | benzene | 12 | 7 | 5 | 0.8333 | 6 | 4 | 1 | 1 |
|  | 4 | phenol | 5 | 4 | 1 | 0.8000 | 3 | 1 | 0 | 1 |
|  | 5 | nitrosamine | 13 | 13 | 0 | 0.5385 | 7 | 0 | 0 | 6 |
|  | 6 | amide | 13 | 12 | 1 | 0.3846 | 4 | 1 | 0 | 8 |
|  | 7 | urea | 8 | 8 | 0 | 0.3750 | 3 | 0 | 0 | 5 |
| **Breast** | 0 | aromatic amine | 8 | 8 | 0 | 0.8750 | 7 | 0 | 0 | 1 |
|  | 1 | primary amine | 13 | 12 | 1 | 0.7692 | 9 | 1 | 0 | 3 |
|  | 2 | alcohol | 13 | 12 | 1 | 0.7692 | 9 | 1 | 0 | 3 |
|  | 3 | benzene | 50 | 36 | 14 | 0.7400 | 25 | 12 | 2 | 11 |
|  | 4 | phenol | 13 | 11 | 2 | 0.6923 | 7 | 2 | 0 | 4 |
|  | 5 | nitro | 13 | 9 | 4 | 0.6154 | 4 | 4 | 0 | 5 |
|  | 6 | halogenated | 26 | 8 | 18 | 0.5769 | 0 | 15 | 3 | 8 |
|  | 7 | ketone | 13 | 11 | 2 | 0.5385 | 5 | 2 | 0 | 6 |
|  | 8 | ether | 8 | 6 | 2 | 0.5000 | 3 | 1 | 1 | 3 |
|  | 9 | tertiary amine | 19 | 15 | 4 | 0.4737 | 5 | 4 | 0 | 10 |
|  | 10 | heteroaromatic | 10 | 8 | 2 | 0.4000 | 2 | 2 | 0 | 6 |
|  | 11 | ester | 14 | 10 | 4 | 0.3571 | 1 | 4 | 0 | 9 |
|  | 12 | amide | 22 | 17 | 5 | 0.3182 | 2 | 5 | 0 | 15 |
|  | 13 | urea | 9 | 7 | 2 | 0.2222 | 0 | 2 | 0 | 7 |
| *Threshold: liver = 0.3210, lung = 0.3656, stomach = 0.3518, breast = 0.3206 | | | | | | | | | | |
